# Supplementary material for: The Association of Selected GWAS Reported AD Risk Loci with CSF Biomarker Levels and Cognitive Decline in Slovenian Patients
Source: Int J Mol Sci. 2023 Aug 19;24(16):12966. doi: 10.3390/ijms241612966 (PMC10455613; doi:10.3390/ijms241612966)
Supplement: Supplementary file 1 [file ijms-24-12966-s001.zip › ijms-2545179-supplementary.pdf]

Table S1: Genotype frequencies of selected polymorphisms.

Gene	SNP	Role	Genotype	All subjects, n = 145 N (%)	MAF	pHWE
SORCS1	rs1358030	g.108123599G>A	CC	17 (11.7)	0.34	0.970
			CT	65 (44.8)		
			TT	63 (43.4)		
	rs1416406	g.109044871A>G	TT	15 (10.3)	0.28	0.163
			TC	52 (35.9)		
			CC	78 (53.8)		
BCHE	rs1803274	p.Ala567Thr	CC	97 (66.9)	0.18	0.709
			CT	44 (30.3)		
			TT	4 (2.8)		
	rs1799807	p.Asp98Gly	TT	139 (95.9)	0.02	0.798
			TC	6 (4.1)		
			CC	0		
TOMM40	rs2075650	g.44892362A>G	AA	80 (55.2)	0.26	0.655
			AG	54 (37.2)		
			GG	11 (7.6)		
	rs157581	p.Phe113=	TT	58 (40)	0.36	0.717
			TC	69 (47.6)		
			CC	18 (12.4)		
ACE	rs1800764	g.63473168C>T	CC	29 (20)	0.44	0.688
			CT	69 (47.6)		
			TT	47 (32.4)		
	rs4343	p.Thr776=	GG	42 (29)	0.48	0.286
			GA	66 (45.5)		
			AA	37 (25.5)		
IL6R	rs2228145	p.Asp358Ala	AA	60 (41.4)	0.34	0.284
			AC	71 (49)		
			CC	14 (9.7)		

Table S2: Association of investigated polymorphisms with cerebrospinal fluid biomarkers among patients with AD.

SNP	Genotype	Aβ	P	$A\beta_{42/40}$ ratio	P	Total tau	P	pTau	P
SORCS1 rs1358030	CC	604 (545-745)	0.504	0.05 (0.04-0.0575)	0.210	1055 (787.75-1289.75)	0.064	130 (95.75-154.5)	0.237
	CT	696 (582.75-773.75)		0.06 (0.04-0.06)		768 (541.5-953)		101.5 (81.75-123.5)	
	TT	688.5 (516.25-766.75)		0.06 (0.05-0.07)		735 (522.5-957)		95 (78-123.75)	
	CT + TT	692 (538.25-769.75)	P_{dom}= 0.497	0.06 (0.04-0.06)	P_{dom}= 0.295	749 (524.25-955.5)	P_{dom}= **0.019**	97 (81-123.75)	P_{dom}= 0.113
SORCS1 rs1416406	TT	697 (565.5-795.5)	0.868	0.06 (0.04-0.07)	0.772	814 (663.5-907.5)	**0.013**	101 (78-120)	**0.036**
	TC	663.5 (531.75-762.25)		0.06 (0.04-0.07)		597.5 (492.75-783.5)		90 (78.5-110.25)	
	CC	689 (534.5-772)		0.05 (0.04-0.06)		855 (557.5-1085.5)		111 (86.5-132.5)	
	TC + CC	669 (538-769)	P_{dom}= 0.609	0.06 (0.04-0.06)	P_{dom}= 0.969	763 (525-1001)	P_{dom}= 0.640	97 (81-126)	P_{dom}= 0.738
BCHE rs1803274	CC	697 (543-770)	0.968	0.05 (0.04-0.06)	0.087	773 (542-921)	0.956	98 (81-122)	0.648
	CT	658 (539-769)		0.06 (0.04-0.07)		778 (496-1151)		101 (79-140)	
	TT	651 (507-.)		0.08 (0.07-.)		735 (722-.)		94.5 (93-.)	
	CT + TT	658 (538.5-777)	P_{dom}= 0.802	0.06 (0.04-0.07)	P_{dom}= 0.290	748 (499-1122.5)	P_{dom}= 0.900	98 (80-135.5)	P_{dom}= 0.482
BCHE rs1799807	TT	668 (538.25-769.75)	0.701	0.05 (0.04-0.06)	0.087	771 (533-968.5)	0.893	98 (81-124)	0.942
	TC	691.5 (607.75-786.5)		0.065 (0.06-0.07)		820 (407.5-1168)		107.5 (59.25-149.75)	
	TC + CC	691.5 (607.75-786.5)	P_{dom}= 0.701	0.065 (0.06-0.07)	P_{dom}= 0.087	820 (407.5-1168)	P_{dom}= 0.893	107.5 (59.25-149.75)	P_{dom}= 0.942
TOMM40 rs2075650	AA	695 (540-767.5)	0.902	0.06 (0.04-0.06)	0.154	766 (543.75-955.5)	0.410	95.5 (81-125.25)	0.449
	AG	655.5 (553.5-770.5)		0.06 (0.05-0.07)		749 (500.5-1004)		97.5 (80-124.25)	
	GG	616.5 (498-799.75)		0.05 (0.03-0.05)		866 (733.25-1203.5)		118 (95.75-151)	
	AG + GG	655.5 (540-772.75)	P_{dom}= 0.784	0.05 (0.04-0.07)	P_{dom}= 0.879	778 (507.5-1010)	P_{dom}= 0.993	101 (81.75-124.75)	P_{dom}= 0.697
TOMM40 rs157581	TT	695 (540-781.25)	0.967	0.06 (0.04-0.06)	0.580	741 (550.75-980.5)	0.405	95.5 (81.75-128)	0.370
	TC	650.5 (544-758.25)		0.06 (0.05-0.06)		761.5 (523.5-912.75)		97.5 (78.5-120)	
	CC	703 (519.75-789.75)		0.05 (0.04-0.06)		866 (664.25-1168.75)		118 (90.75-142.75)	
	TC + CC	655.5 (540-766.5)	P_{dom}= 0.984	0.06 (0.05-0.06)	P_{dom}= 0.564	780 (524.25-993)	P_{dom}= 0.922	99 (81-123.75)	P_{dom}= 0.988

ACE rs1800764	CC	587.5 (538.25-770)	0.741	0.05 (0.04-0.06)	0.997	758.5 (524.25-958.25)	0.636	97.5 (82.75-124)	0.786
	CT	708.5 (560-785.75)		0.06 (0.04-0.06)		770.5 (556.5-1024.75)		99 (84-132.5)	
	TT	652.5 (537.75-750.25)		0.06 (0.04-0.07)		777.5 (490.5-888.5)		99 (80-116)	
	CT + TT	696 (546.25-769.75)	P_{dom}= 0.718	0.06 (0.04-0.06)	P_{dom}= 0.937	775.5 (543.75-993)	P_{dom}= 0.652	99 (81-127.25)	P_{dom}= 0.899
ACE rs4343	GG	692 (538.75-762)	0.545	0.06 (0.0475-0.0625)	0.535	735 (524.25-873.5)	0.395	97.5 (81-124)	0.880
	GA	692.5 (564-785.75)		0.06 (0.04-0.06)		782.5 (546.5-1052.75)		97.5 (83.25-126.5)	
	AA	598.5 (516-738)		0.045 (0.03-0.0675)		793.5 (484.25-992.5)		103 (81-134.75)	
	GA + AA	668 (539.75-771.25)	P_{dom}= 0.878	0.055 (0.04-0.06)	P_{dom}= 0.533	782.5 (537-1014.75)	P_{dom}= 0.216	99.5 (81-128.75)	P_{dom}= 0.618
ACE2 rs1978124	TT	713.5 (545-799.75)	0.201	0.06 (0.04-0.07)	0.124	733.5 (543.75-859.25)	0.106	96.5 (81-121.25)	0.217
	TC	636 (520-744.5)		0.05 (0.0375-0.06)		872.5 (563-1168.75)		116.5 (83.25-151)	
	CC	643 (562-750)		0.055 (0.0475-0.07)		747.5 (511.75-973)		99 (76.75-117.5)	
	TC + CC	643 (532-745.25)	P_{dom}= 0.088	0.05 (0.04-0.06)	P_{dom}= 0.208	810 (526.5-1034.25)	P_{dom}= 0.139	101.5 (81-127.5)	P_{dom}= 0.321
ACE2 rs2285666	CC	669 (518-769.5)	0.433	0.05 (0.04-0.06)	0.374	778 (545.5-993)	0.859	98 (81-124.5)	0.809
	CT	614 (575-775)		0.06 (0.04-0.06)		722 (515-967)		93 (77-137)	
	TT	708 (652.75-832.5)		0.07 (0.05-0.07)		685.5 (495-1021.5)		104 (71-113)	
	CT + TT	695 (585-775)	P_{dom}= 0.345	0.06 (0.04-0.06)	P_{dom}= 0.508	722 (515-969)	P_{dom}= 0.646	100 (77-128)	P_{dom}= 0.525
IL6R rs2228145	AA	595 (513-774.5)	0.120	0.05 (0.04-0.065)	0.900	773 (552.5-1085.5)	0.446	102 (82.5-129.5)	0.542
	AC	711 (581-788)		0.06 (0.04-0.06)		748 (522-911)		97 (81-123)	
	CC	592 (495-685.75)		0.06 (0.035-0.0675)		838.5 (541.5-1147)		99.5 (73.75-145.25)	
	AC + CC	695 (570-760)	P_{dom}= 0.400	0.06 (0.04-0.06)	P_{dom}= 0.646	763 (524-921)	P_{dom}= 0.425	97 (81-124)	P_{dom}= 0.269

Table S3: Association of investigated polymorphisms with cognitive test scores among all patients.

SNP	Genotype	MMSE	P
SORCS1 rs1358030	CC	26.5 (21-27.25)	0.425
	CT	26 (24-27)	
	TT	25.5 (21.75-26.75)	
	CT + TT	26 (24-27)	P_{dom}=0.643
SORCS1 rs1416406	TT	23 (20.25-26)	0.898
	TC	26 (23.75-26.25)	
	CC	26 (25-27)	
	TC + CC	26 (24-27)	P_{dom}=0.748
BCHE rs1803274	CC	26 (24-27)	0.056
	CT	24 (21-26)	
	TT	21.5 (16-.)	
	CT + TT	24 (21-26)	P_{dom}=**0.029**
TOMM40 rs2075650	AA	26 (24-27)	0.484
	AG	26 (22-27)	
	AG + GG	26 (22.5-27)	P_{dom}=0.608
TOMM40 rs157581	TT	26 (24.75-27)	0.318
	TC	26 (21.5-27)	
	CC	26 (16-.)	
	TC + CC	26 (21-27)	P_{dom}=0.260
ACE rs1800764	CC	24.5 (21-26)	0.197
	CT	26 (24-27.75)	
	TT	26 (24.5-27)	
	CT + TT	26 (24-27)	P_{dom}=0.077
ACE rs4343	GG	26 (23-26)	0.463
	GA	25 (23-27.5)	
	AA	26 (25-27)	
	GA + AA	26 (23.75-27)	P_{dom}=0.729
ACE2 rs1978124	TT	26 (24-27)	0.785
	TC	26 (22.25-28.25)	

	CC	26 (22.5-26.5)	
	TC + CC	26 (23-27)	P_{dom}=0.983
ACE2 rs2285666	CC	26 (24-26.75)	0.495
	CT	26 (23-27.75)	
	TT	26 (22.5-27)	
	CT + TT	26 (23-27)	P_{dom}=0.624
IL6R rs2228145	AA	26 (24-27)	0.841
	AC	25.5 (21-27)	
	CC	25 (23.25-26)	
	AC + CC	25.5 (22.5-26.25)	P_{dom}=0.591
